# Supplementary material for: Multimodal machine learning for distinguishing pediatric multiple sclerosis from non-inflammatory conditions using optical coherence tomography
Source: Front Neurol. 2026 Apr 21;17:1791696. doi: 10.3389/fneur.2026.1791696 (PMC13138958; doi:10.3389/fneur.2026.1791696)
Supplement: Supplementary file 1 [file Supplementary_file_1.pdf]

Supplementary Material

Appendix 1 Diagnoses in the Non-inflammatory Cohort

The non-inflammatory cohort comprised individuals with diverse neurological and systemic conditions. The most frequent diagnosis was Langerhans Cell Histiocytosis (LCH). Other diagnoses included spinal cord infarct, stroke, astrocytoma, recurrent facial palsy without brain lesions, headache with nonspecific white matter (WM) abnormalities, hallucinations, tingling with normal brain MRI, oral ulcers without a neurological diagnosis, functional visual loss with normal exams, nonspecific cord abnormalities, and WM abnormalities including glioma. Several individuals also exhibited nonspecific white matter changes on MRI without a definitive neurological diagnosis.

Table 1 Diagnoses in the non-inflammatory cohort. This table summarizes the alternative neurological and systemic conditions identified among patients without inflammatory demyelinating disease.

**Diagnosis**	**Number of Patients**
Langerhans Cell Histiocytosis (LCH)	13
Spinal cord infarct	1
Stroke	1
Astrocytoma	1
Recurrent facial palsy (no brain lesions)	1
Headache with nonspecific WM abnormalities	3
Hallucinations with nonspecific WM abnormalities	2
Nonspecific white matter abnormalities	4
Nonspecific cord abnormality	1
White matter abnormalities – glioma	1
Tingling, brain MRI normal, no lesions	1

Oral ulcers, no neurological diagnosis	1
Functional visual loss, normal exam	1

Appendix 2 OCT Features List

Table 2 Description of Optical Coherence Tomography (OCT) Features.

Feature	Eye
**Retinal Nerve Fiber Layer (RNFL)**	
**RNFL Thickness**	Right/Left
**Superior RNFL Thickness**	Right/Left
**Nasal RNFL Thickness**	Right/Left
**Inferior RNFL Thickness**	Right/Left
**Temporal RNFL Thickness**	Right/Left
**Ganglion Cell Layer (GCL)**	
**GCL Thickness – Superior**	Right/Left
**GCL Thickness – Superotemporal**	Right/Left
**GCL Thickness – Inferotemporal**	Right/Left
**GCL Thickness – Inferior**	Right/Left
**GCL Thickness – Inferonasal**	Right/Left
**GCL Thickness – Superonasal**	Right/Left
**GCL – Average Thickness**	Right/Left
**GCL – Minimum Thickness**	Right/Left
**Macular Thickness (MT)**	
**Macular Thickness – Superior (Ring 0)**	Right/Left
**Macular Thickness – Nasal (Ring 0)**	Right/Left
**Macular Thickness – Inferior (Ring 0)**	Right/Left
**Macular Thickness – Temporal (Ring 0)**	Right/Left
**Macular Thickness – Superior (Ring 1)**	Right/Left
**Macular Thickness – Nasal (Ring 1)**	Right/Left

**Macular Thickness – Inferior (Ring 1)**	Right/Left
**Macular Thickness – Temporal (Ring 1)**	Right/Left
**Central Subfield Thickness**	Right/Left
**Macular Cube Volume**	Right/Left
**Macular Cube Average Thickness**	Right/Left
**Foveal Features**	
**Foveal Measurement 1**	Right/Left
**Foveal Measurement 2**	Right/Left

Appendix 3 Preprocessing Pipeline

An overview of the full preprocessing pipeline is presented in Figure 1.

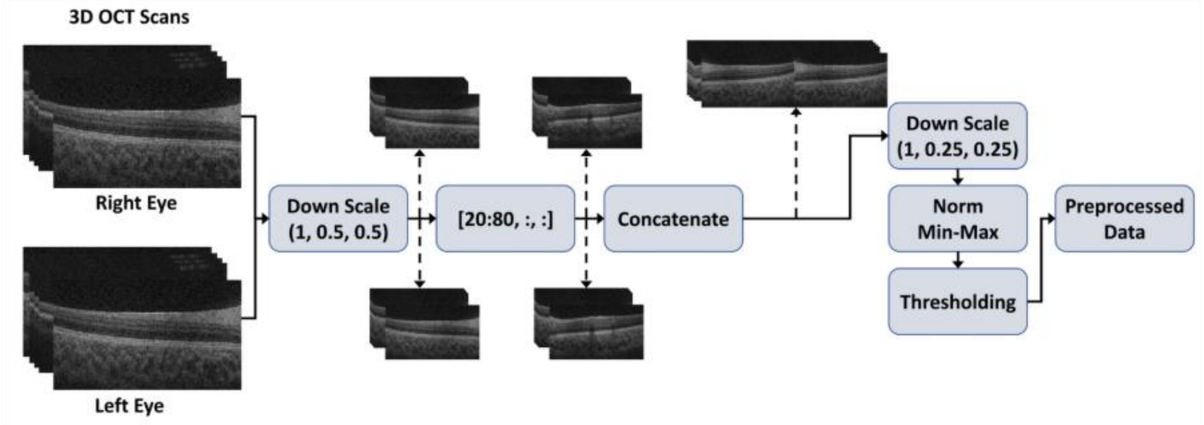

Supplementary Figure 1 Overview of the preprocessing pipeline.

Appendix 4 Baseline OCT Metrics in MS and Non-Inflammatory Groups

Table 3 presents the average and standard deviation of selected OCT-derived parameters for both MS and non-inflammatory groups. These include global and regional retinal layer thicknesses, foveal and ganglion cell layer measurements, as well as clinically relevant inter-eye differences and RNFL thinning metrics commonly associated with neurodegeneration in MS. To compute these statistics, we include only the first available (baseline) scan per patient.

Table 3 Summary of OCT features for MS and Non-Inflammatory Groups. Values are reported as mean ± standard deviation or count (percentage) as appropriate.

OCT Parameter / Statistic	*MS (n = 211)*	*Non-inflammatory (n = 52)*
**Retinal Layer Thickness (in μm)**		
RNFL Thickness	93.55 ± 13.85	98.53 ± 6.30
Superior Quadrant Thickness	121.59 ± 18.88	127.34 ± 10.58
Nasal Quadrant Thickness	70.72 ± 12.13	72.72 ± 10.70
Inferior Quadrant Thickness	121.81 ± 22.70	126.14 ± 12.65
Temporal Quadrant Thickness	60.05 ± 15.01	68.34 ± 9.98
Foveal Thickness (Inner)	253.44 ± 7.85	253.55 ± 10.94
Foveal Thickness (Outer)	66.33 ± 3.43	67.22 ± 2.85
Ganglion Cell Layer Thickness (GCL)	76.76 ± 9.48	85.34 ± 4.16
**Clinical Threshold-Based Metrics**		
Inter-Eye RNFL Difference (μm)	8.84 ± 15.47	3.34 ± 2.79
# Inter-Eye RNFL Difference ≥ 6	29 (35%)	4 (13%)
# RNFL Thickness ≤ 87	23 (28%)	2 (6%)

Appendix 5 OCT Imaging Data and Retinal Layer Annotation

Supplementary Figure 1 shows a representative B-scan slice from a macular OCT acquisition, illustrating the retinal layers captured by the Cirrus HD-OCT Macular Cube (512×128) protocol. The image depicts the foveal depression and annotates the key layers of clinical relevance in MS: the retinal nerve fiber layer (RNFL), ganglion cell layer (GCL), inner plexiform layer (IPL), and inner nuclear layer (INL). These layers are automatically segmented by the device software to generate quantitative thickness measurements, including global and quadrant-level RNFL thickness and six sectoral GCIPL measurements, which together constitute the 52 tabular features used alongside the raw 3D OCT volumes in our multimodal classification framework.

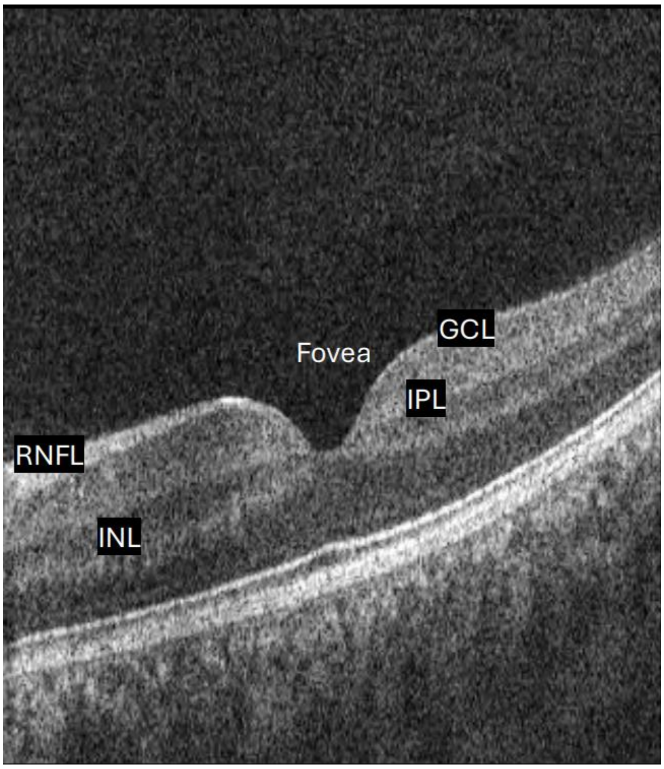

Supplementary Figure 2 Representative macular OCT cross-section with annotated retinal layers. A single B-scan slice from a Macular Cube (512×128) acquisition obtained using the Cirrus HD-OCT scanner (Carl Zeiss Meditec, Model 5000), centered on the macula and showing the characteristic foveal depression. Key retinal layers relevant to MS-associated neurodegeneration are annotated: the retinal nerve fiber layer (RNFL), ganglion cell layer (GCL), inner plexiform layer (IPL), and inner nuclear layer (INL). The fovea is indicated as the anatomical reference point. Quantitative thickness measurements of the RNFL and the combined ganglion cell–inner plexiform layer (GCIPL) derived from these regions form the basis of the 52 tabular OCT features used as one of the two input modalities in this study.
